# Prevalence and predictors of atypical histology in endometrial polyps removed by hysteroscopy: A secondary analysis from the SICMIG hysteroscopy trial

**Published:** 2019-06

**Authors:** For the Italian School of Minimally Invasive GynecologicaL Surgery (SiCMiG) Hysteroscopists Group, G Garuti, M Luerti, FPG Leone, G Perrini, D Dealberti, V Vitelli, S Angioni, A Vitagliano, A Di Spiezio Sardo, G Benassi, C De Angelis, L Nappi, S Bettocchi, P Casadio, GL Marchino, G Lanzo, E Busato, S Calzolari, E Castellacci, G Giarrè, C Personeni, F Mangino, F Scrimin, V Cela, P Florio

**Affiliations:** Department of Obstetrics and Gynecology, Lodi Hospital, Italy;; Department of Gynecology, Istituto Clinico Città Studi, Milan, Italy;; Department of Obstetrics and Gynecology, DSC L. Sacco, Milan, Italy;; Department of Gynecology, Mauriziano Umberto I Hospital, Turin, Italy;; Department of Obstetrics and Gynecology, SS. Antonio e Biagio e Cesare Arrigo Hospital, Alessandria, Italy;; Department of Biostatistics, Oslo Center for Biostatistics and Epidemiology, University of Oslo, Norway;; Department of Obstetrics and Gynecology, University of Cagliari, Italy;; Department of Women’s and Children’s Health, University of Padua, Italy;; Department of Obstetrics and Gynecological, Urological Sciences and Reproductive Medicine, University “Federico II” of Naples, Italy;; Department of Minimally Invasive and Oncological Surgery, Casa di cura Città di Parma, Italy;; Department of Women’s Health and Territorial Medicine, University of Rome “La Sapienza”, Rome, Italy;; Department of Medical and Surgical Sciences, University of Foggia, Italy;; Department of Obstetrics , Gynecology and Neonatology, University of Bari, Italy;; Department of Obstetrics and Gynecology, University of Bologna, Italy;; Department of Obstetrics and Gynecology, University of Turin, Italy;; Department of Obstetrics and Gynecology, Treviso Hospital, Treviso, Italy;; Department of Day Surgery, Palagi Hospital, Florence, Italy;; Institute for Maternal and Child Health—IRCCS “Burlo Garofolo”, Trieste, Italy;; Department of Obstetrics and Gynecology, University of Pisa, Italy;; Department of Obstetrics and Gynecology, Pistoia Hospital, Italy.

**Keywords:** Endometrial polyp, Atypical hyperplasia, Endometrial cancer, Hysteroscopy, Polypectomy

## Abstract

**Background:**

The aim of this study is to assess the prevalence of atypical hyperplasia (AH) and endometrial cancer (EC) within endometrial polyps (EPs) removed by hysteroscopy.

**Methods:**

Hysteroscopic polypectomy interventions were performed over 1436 consecutive patients with Eps to complete a prospective observational trial (Canadian Task Force Classification II-2) including 19 Italian Gynecologic Departments (University-Affiliated or Public Hospitals) for a secondary multicenter analysis.

**Results:**

At histological analysis, in 1404 patients (97.8%) EPs were classified as benign, whereas in 32 patients (2.2%) EPs were diagnosed as atypical (i.e. with AH or EC). Specifically, AH and EC were found in 17 (1.2%) and 15 (1.0%) cases, respectively. Risk factor analysis showed that menopausal status, BMI and size of EPs were associated with increased risk of atypical EPs (p<0.0001). Abnormal uterine bleeding, EPs number, contraceptive therapy and tamoxifen were not associated with increased risk of atypia (p=ns). The cut-off points for increased risk of atypical polyps were 54.2 years old, BMI of 25.3 and EP size of 2.2 cm. Hysterectomy specimens were analyzed in 21 women with atypical EPs, showing the concomitant presence of atypical tissue in non-polypoid endometrium in the majority of patients (n=14 women, 66.6%).

**Conclusion:**

The prevalence of endometrial cancer and atypical hyperplasia in endometrial polyps is low, although it is increased in women who are overweight, older than 54 years of age or with a polyp larger than 2cm.

## Introduction

Endometrial polyps (EPs) are sessile or pedunculated focal mucosal projections, arising as monoclonal overgrowth of genetically altered stromal cells, supplied by a thick vascular stalk and covered by secondly-induced surface epithelium and glands (WHO Classification of Tumors of Female Reproductive Organs, 2014).

EPs represent the most commonly encountered endometrial lesions, with a reported prevalence of 8% to 35% and increasing frequency with age. Likewise, women with EPs can be asymptomatic or suffer from abnormal uterine bleeding (AUB) (Sahrma et al., 2004; Dreisler et al., 2009; Lieng et al., 2009; Practice Committee of the AAGL, 2012). AUB is the most common symptom of EPs, occurring in 10% to 40% of patients (Clevenger-Hoeft et al., 1999; Anastasiadis et al., 2000;). Moreover, EPs can be found in up to 30% of infertile women (Hinckley and Milki, 2004), although a causal relationship between EPs and infertility was demonstrated by a single randomized-controlled trial (Perez-Medina et al., 2005). Based on the above, practice guidelines recommend the surgical removal of EPs for symptomatic women and in case of infertility (Practice Committee of the AAGL, 2012).

Importantly, EPs may sometimes exhibit atypical cells at histological examination. The prevalence of Atypical Hyperplasia (AH) and Endometrial Cancer (EC) ranges from 0.5% to 13% in women with EPs (Hileeto et al., 2005; Machtinger et al., 2005). Previous studies found that the risk of discovering atypical histology within EPs can vary depending on patients’ age, menopausal status, AUB, EPs size and number, hypertension, obesity, diabetes or tamoxifen intake (Savelli et al., 2003; Ben-Arie et al., 2004; Lieng et al., 2007; Ferrazzi et al., 2009). Nevertheless, the body of evidence on this topic is poor and the management of EPs is still subject to an individual approach in asymptomatic patients (taking into account women’s preferences).

Recently, we conducted a multicenter trial evaluating the effectiveness of inpatient vs outpatient hysteroscopy for achieving a complete removal of EPs on a large population in which polypectomy was indicated (n=1519 patients) (Luerti et al., 2018). As a secondary analysis of this trial, we aimed to estimate the prevalence and risk factors of atypical histology (AH and EC) within the EPs removed.

## Materials and methods

### Study design

This is a secondary analysis of the SICMIG hysteroscopy trial (Luerti et al., 2018), conducted between January and December 2016 in 19 Italian hysteroscopy Units (University-Affiliated or Public Hospitals). All surgeons involved in the study were certified as experienced in hysteroscopic surgery by the Italian School of Minimally Invasive Gynecologic Surgery (SICMIG). The study was exempt from institutional review board (IRB) approval because its design was observational (i.e. without any modification of the routine clinical practice in each center) and all data was anonymized before analysis.

### Patients

We recruited a consecutive series of patients with a sonographic diagnosis of EPs in whom hysteroscopic polypectomy was indicated.

In all patients, a transvaginal sonography (TVS) was performed before hysteroscopy, according to the IETA protocol (Leone et al., 2010). In pre-menopausal women, TVS was performed in the early proliferative phase (day cycle 4 – 6), whilst in postmenopausal women on cyclic hormonal therapy TVS was performed 5 – 10 days following the last progestin tablet. The diagnosis of EP was suspected when finding either a focal uniform hyperechogenic area > 10 mm, or a non-uniform area, due to the presence of cystic areas. Occasionally, it was recognized “the bright edge” sign, which is the echo formed by the interface between an intracavitary lesion and the endometrium. Color-Doppler allowed in some cases the recognition of a single dominant vessel, with or without branching, entering into the lesion. In case of doubtful images at TVS, a sonohysterography was performed in order to better characterize EPs before hysteroscopy.

EPs size was evaluated with one or more of the following methods:

- by measuring the largest diameter obtained in the longitudinal and transverse ultrasonographic scans of the uterine cavity.
- by hysteroscopy-view comparison between the length of surgical devices used for EP removal (i.e. opening of the jaws of 5Fr/7Fr grasping forceps or scissors, tip of co-axial microelectrodes and size of the resectoscopic loops) and the largest diameter of the polyp
- by direct measurement of the largest diameter of the lesion after its “en-bloc” retrieval.

After patients’ counseling, either an out-patient or in-patient polypectomy was planned, based on clinical background, Institutional customs and patient’s preference. Women were considered post-menopausal if they reported a period of amenorrhea of at least 12 months after an age of 45 years. AUB was defined as any vaginal bleeding in post-menopausal women. In fertile women AUB was defined accordingly with the 2011 FIGO classification as acute periodic heavy menstrual bleeding or inter-menstrual bleeding lasting less than 6 months (Munro et al., 2011).

### Surgical techniques

All procedures were assisted by video-camera and were conducted by using a fluid distending medium delivered by pressure bag or a peristaltic pump. Normal saline or hypotonic solutions were used as distension medium, depending on the type of adopted instrumentation. Polypectomy was carried-out by using one of the following hysteroscopes: i. Double-flow 12Fr-16Fr rigid hysteroscopes with 5Fr operative channels ii. 26Fr-27Fr resectoscopes iii. 16Fr mini-resectoscope iv. Double-flow 10Fr fiber-based hysteroscope with 7Fr operative channel v. 13Fr hysteroscopy morcellation system.

The cutting devices used for EPs removal included: i. Mechanical tools such as sharp scissors, grasping forceps and morcellator ii. Electrosurgical tools such as 5Fr co-axial electrodes, bipolar or monopolar loops, miniloops iii. Polyfiber diode laser probes.

### Surgery

Based on hysteroscopy-view EPs were defined as focal, single or multiple sessile or pedunculated luminal projections, fluttering under the distending medium flow, showing from soft to mild-fibrous consistence, covered by an evenly lined functional or atrophic mucosa, frequently showing cyst-gland formation and supplied by a thin vascular network (Di Spiezio et al., 2015). Depending on anatomic characteristics of EPs and surgical devices used, either an “en-bloc” or a “slicing” polypectomy inclusive of the polyp’s pedicle was accomplished. All interventions were carried-out as a single procedure; the collected specimens underwent histopathology assessment and pathologic reports have been supplied according to the WHO current classification (WHO Classification of Tumors of Female Reproductive Organs, 2014).

### Histological analysis

All the EPs specimens were reviewed by a single gynaecological pathologist per center with specialized training in gynecologic pathology. The most significant pathologic diagnosis was recorded for each subject. Endometrial and EPs pathologies were defined as benign, non-atypical (simple or complex) hyperplasia, atypical hyperplasia or cancer using standard pathologic criteria. Endometrial hyperplasia and cancer were characterized as arising in the EP or the adjacent endometrium.

### Data collection

A single physician per center collected data about the general features of patients (including age, menopausal status, body mass index [BMI], history of AUB, therapy with tamoxifen or contraceptives, EPs number, EPs size) and the results of histological analysis on EPs. Based on histopathology, EPs were categorized as “common EPs” and “atypical EPs”. Common EPs included all the cases of EPs without atypical histology (including non-atypical hyperplasia). Atypical EPs included those EPs with AH or EC.

In the patients showing either an AH or an EC confined to an EP and who underwent hysterectomy, the available histopathology findings obtained from hysterectomy specimens were recorded and discussed.

### Study outcomes

The primary outcome was to assess the prevalence of atypical EPs in the study population. Secondary outcome was the evaluation of the risk factors for atypical EPs.

### Statistical analysis

Descriptive statistics of demographic and clinical characteristics are reported in Table I. The statistical association of these demographic and clinical characteristics to the atypical histology (AH and infiltrating EC) was tested differently according to the variable type. For continuous variables (age, BMI, EP size), we performed a Mann-Whitney test to assess the difference in mean of each variable in the two groups (non-atypical and atypical pathology). For categorical variables (menopausal status, tamoxifen intake, estroprogestins intake, AUB, polyp number), we performed a chi-squared test of association, where we computed a simulated p-value to gain power given the small proportion of atypical histology on the total of EP. P-values of both tests are reported in Table III. Note that since we are performing these tests of associations on 11 possibly correlated clinical variables, we have to correct for multiple testing to be able to draw the test conclusions jointly. Multiple testing was accounted for via Bonferroni correction, so that the corrected level of significance of the tests is 0.05/11 = 0.0045. A multivariate logistic model has also been used for estimating the odds ratio associated to each demographic and clinical characteristics when predicting the atypical histology. The best multivariate logistic regression model has been estimated including age, menopausal status, BMI and the polyp dimension as significant predictors.

**Table I:** — General demographic characteristics of included patients.

Variable		Number (%) or mean (± SD)
Age (years)		
	Mean (SD)	52.44 (12.63)
	95% CI (Gaussianity assumption met, t-student CI)	[51.81; 53.08]
Body Mass Index (kg/m^2^)		
	Median	24.065
	Range	[15.0; 51.4]
AUB		
	Heavy menstrual bleeding	174 (11.05%)
	Intermenstrual bleeding	205 (13.02%)
	Postmenopausal bleeding	302 (19.19%)
Menopausal status		
	Premenopause	689 (43.77%)
	Postmenopause	805 (51.14%)
Current tamoxifen intake		
	No	1481 (97.5%)
	Yes	38 (2.5%)
Current estroprogestins intake (OCs, HRT, Tibolone)		
	No	1467 (96.5%)
	Yes	52 (3.42%)
Polyp’size (mean polyp size 17.61 mm [SD 9.4])		
	>10 mm <15 mm	578 (38.05 %)
	>15 mm <20 mm	416 (27.39%)
	>20 mm	525 (34.56%)
Polyp number		
	One polyp	1175 (77.35%)
	More than 1 polyp	344 (22.64%)

Note that n=838 women (53.24%) were asymptomatic at the time of polyp diagnosis.

**Table III:** — Descriptive statistics for all clinical variables.

Clinical Variable	Descriptive Statistics	p-value
Age	NA 52.3 + 12.6	0
Years, M +SD	A 61.0 + 10.8	
Menopausal status Number of patients (%)	Premenopause NA 647 (46.1 %) A 3 (9.4 %) Post menopause NA 735 (52.3 %) A 29 (90.6 %)	0.0004998
BMI	NA 25.0 + 4.7	0
Kg/m2, M + SD	A 29.1 + 6.0	
Tamoxifen intake	NA 34 (4.4 %)	0.1879
Number of patients (%)	A 2 (6.2 %)	
Estroprogestin intake	NA 48 (3.4%)	0.09839
Number of patients (%)	A 3 (9.3%)	
Bleeding symptoms Number of patients (%)	AUB NA 638 (45.4 %) A 13 (40.6 %) Asymptomatic NA 766 (54.5 %) A 19 (59.4 %)	0.01049
Polyp’size	NA 17.3 + 8.5	0
mm, M + SD	A 24.5 + 24.9	
Polyp number Number of patients (%)	One polyp NA 1086 (77.3 %) A 23 (71.9 %) More than one polyp NA 318 (22.6 %) A 9 (28.1 %)	0.7416

(NA= Non Atypical EP, A=Atypical EP, M= Mean value, SD=Standard Deviation value)

## Results

**Figure 1:**
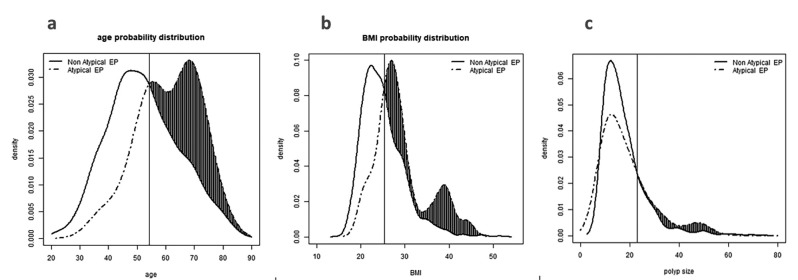
a) Probability distribution of atypical endometrial polyps according to age. The vertical line indicates the age cutoff, and the shaded area the increased risk. b) Probability distribution of atypical endometrial polyps according to BMI. The vertical line indicates the BMI cutoff, and the shaded area the increased risk. c) Probability distribution of atypical endometrial polyps according to size of endometrial polyps. The vertical line indicates the polyp size cutoff, and the shaded area the increased risk.

A total number of 1519 patients underwent hysteroscopic single or multiple polypectomy. Demographic and clinical characteristics of the study population are reported in Table I. In 1404 women, histological analysis confirmed the diagnosis of common EPs (92.4%), including non-atypical hyperplasia in 142 cases (9.3%). In 32 patients (2.22%), histological analysis revealed atypical EPs, with AH and EC in 17 (1.18%) and 15 (1.04%) cases, respectively. All the EC were endometrioid histotypes. In 83 women, benign pathologies other than EP were found at histology. These patients were excluded, resulting in a final study population of 1436 patients. Data on histopathological findings are shown in Table II. Risk factor analysis showed that higher patients’ age (mean age of 61.0 ± 10.8 vs 52.3 ± 12.6 years in atypical vs typical EPs, p<0.0001), BMI (mean BMI of 29.1 ± 6.0 vs 25.0 ± 4.7 in atypical vs typical EPs, p<0.0001) and size of EPs (mean EPs size of 24.5 ± 24.9 vs 17.3 ± 8.5 in atypical vs typical EPs, p<0.0001) were significantly associated with increased risk of atypical EPs. The smallest values of age, BMI and EP size associated with atypical EPs were respectively 54.2 years, 25.3 Kg/m2 and 22.9 mm (Figure 1). Furthermore, we found higher risk of atypical EPs in post-menopausal patients compared to pre-menopausal patients (29/764 vs 3/650, p=0.0005). The use of a multivariate logistic regression model confirmed these results: menopausal status, BMI and the polyp dimension are all significant factors for predicting the atypical nature of the polyp. Precisely, they all increase the risk of the patients even when adjusting for the possible confounding of the others. However, the use of a multivariate model for our data poses issues, due to the limited number of patients (only 21) having atypical polyps: the power of the tests is heavily reduced in this case, and it is better to only rely on the univariate chi-squared results.

**Table II:** — Histopathological diagnoses obtained on 1519 hysteroscopic polypectomies.

Histopathological diagnosis	Number (%)
Typical polyp	1262 (83.08%)
Polyp with non atypical hyperplasia	142 9.34%)
Polyp with atypical hyperplasia	17 (1.1%)
Polyp with infiltrating carcinoma	15 (0.99%)
Myoma	18 (1.18%)
Functional endometrium	29 (1.91%)
Non atypical hyperplasia	12 (0.79%)
Cystic atrophy	17 (1.12%)
Other	7 (0.46%)

Differently, no significant correlation was found between bleeding symptoms, estroprogestogens intake, tamoxifen therapy, EPs number and increased risk of atypical EPs (p=ns). For 21 out of the 32 patients with atypical EPs, the pathological report of hysterectomy was available. In 14 (66.6%) of these women, synchronous ECs (n=10 cases) or AHs (n=4 cases) were found also in surrounding non-polypous endometrium. All data are summarized in Table IV.

**Table IV:** — Pathologic reports obtained in 21 hysterectomy specimens available, afterward an atypical polyp diagnosed in hysteroscopic resection products.

Polyp Pathology (Number)	Hysterectomy Pathology of Surrounding Endometrium (number)
Polyps with Atypical Hyperplasia (9)	Atypical Hyperplasia (3) Endometrial Carcinoma (1) Normal Endometrium (5)
Polyps with Endometrial Carcinoma (12)	Atypical Hyperplasia (1) Endometrial Carcinoma (9) Normal Endometrium (2)

## Discussion

With the advent of miniaturized hysteroscopes and the improvement of the surgical techniques, hysteroscopy has become the gold-standard technique for treating EPs, therefore overcoming the limitations of blind procedures (Di Spiezio et al., 2015).

In the pre-hysteroscopic era, atypical histology was reported in association with EPs in up to 30% of cases. We may argue that such high prevalence of atiypa in patients with EPs was probably due to the analysis of curettage material (Peterson and Novak, 1956; Buckley and Fox., 1989; Angioni et al., 2008). After endometrial curettage, all the removed tissue specimens are comminuted together in a unique sample, making it difficult for the pathologist to identify the originating site of atypical tissue (i.e. from EPs, from endometrium or from stand-alone atypical lesions). To establish that an EC or AH has arisen from an EP, is necessary to demonstrate that the atypical tissue is separated from normal endometrium by the base of the EP, this latter showing no malignant features (Wang et al., 2010).

Hysteroscopy allows a selective and complete EP resection (i.e. inclusive of the pedicle of EP), thus minimizing the fragmentation of the lesion and facilitating the histopathological identification of the originating site of atypical tissue. In the last two decades, several retrospective studies reported that discovering malignant cells within hysteroscopically removed EPs is quite uncommon, with a mean incidence of 3.6% (Savelli et al., 2003; Ben-Arie et al., 2004; Shushan et al., 2004; Machtinger et al., 2005; Parra et al., 2006; Antunes et al., 2007; Lieng et al., 2007; Baiocchi et al., 2009; Gregoriou et al., 2009; Perri et al., 2010; Wang et al., 2010; Costa-Paiva et al., 2011; Ricciardi et al., 2014; Elyashiv et al., 2017), ranging from 1.3% (Shushan et al., 2004) to 6.3% (Ben-Arie et al., 2004).

The majority of authors agree that the risk of malignancy is significantly increased in post-menopausal women, with a Relative Risk (RR) of 3.8 compared to premenopausal women and a prevalence of atypical lesions in up to 12.5 % of patients (Lieng et al., 2007; Gregoriou et al., 2009). AUB is considered another strong indicator of malignancy, showing an association with atypical EPs in up to 10.8 % of patients (Baiocchi et al., 2009), with a RR of 1.9 compared to asymptomatic women (Gregoriou et al., 2009). Also, EP size was found to be a risk factor for malignant EPs, where a mean diameter ranging from 10 to 18 mm was considered as a cut-off for potential risk of malignancy (Ben-Arie et al., 2004; Ferrazzi et al., 2009; Wang et al., 2010; Costa-Paiva et al., 2011). Moreover, hypertension was found to be associated with increased risk of malignancy in different studies (Savelli et al., 2003; Baiocchi et al., 2009; Costa-Paiva et al., 2011), as well as BMI and multiple polyps (Parra et al., 2006; Costa-Paiva et al., 2011).

Ours is one of the largest prospective studies evaluating the risk of atypical lesions within EPs. We found a cumulative low prevalence of atypical pathology within apparently normal EPs, which occurred in the 2.22% of the study population. AH and EC showed a similar prevalence in our study (1.18% and 1.04%, respectively), confirming the data from a previous study (Elyashiv et al., 2017). Our results are in line with those from other studies reporting a pooled prevalence of AH and EC in 1.3%-2.4% of patients with EPs (Shushan et al., 2004; Angioni et al.; 2008; Elyashiv et al., 2017).

Moreover, we found a very low prevalence of atypical EPs in premenopausal women (0.46%), in line with other Authors’ findings (Machtinger et al., 2005: Parra et al., 2006; Perri et al., 2010). Conversely, the prevalence of atypical lesions was considerably higher in the subgroup of post-menopausal women (3.79%). Age is a well-known risk factor of EC and this evidence was further confirmed by our study, where statistical analysis determined a cut-off of increased risk of atipya of 54.2 years. This data was in agreement with that by Perri et al. (2010) who identified a cut-off point of 55 years, whereas it was lower than the one of 60 years reported by other authors (Antunes et al., 2007; Ricciardi et al., 2014). Although obesity has been etiologically associated with EC due to higher circulating levels of serum estrogens compared to normal-weight women, the risk of atypical EPs did not correlate with the increase of BMI in different studies (Savelli et al., 2003; Antunes et al., 2007; Lieng et al., 2007; Ricciardi et al., 2014). Only in one study, Costa Paiva et al. (2011) found a significant correlation between obesity (i.e. BMI higher than 30 Kg/m^2^ ) and the risk of EC. In our study, we found a strong correlation between overweight and atypical EPs, with a BMI cut-off of 25.3.

Regarding the risk of malignancy in relation to EPs size, recent studies found increased risk below the cut-off points 1.0-1.8 cm (Ben-Arie et al., 2004; Ferrazzi et al., 2009; Wang et al., 2010; Costa-Paiva et al., 2011). We found a higher cut-off point (2.2. cm) conferring increased risk of EC compared to those studies. At this regard, we may argue that our measurements of EPs could be more accurate than those of other studies, because all the physicians adhered to IETA guidelines.

Another interesting finding of our study was the high percentage of atypical EPs discovered in asymptomatic women (n=19; 59.4% of the total number of atypical EPs). At this regard, is to be noted that more than half of the study population was asymptomatic at the time of polypectomy. This data is not in agreement with that from other studies, where AUB was significantly associated with increased risk of EC (Lee et al., 2010). Conversely, other authors found similar results (Savelli et al., 2003; Ben-Arie et al., 2004; Lieng et al., 2007; Baiocchi et al., 2009).

However, we should stress that the total number of atypical polyps in our study was too low (n=32) to draw firm conclusions. Moreover, the fact that 19 women with atypical polyps were asymptomatic at the time of polypectomy does not exclude the possibility that the same patients would have successively displayed AUB.

Our risk factor analysis showed that previous estroprogestins treatment, tamoxifen intake and EPs were not significantly associated with increased risk of atypical EPs. These results were in line with those from similar studies. Only a single study reported a correlation between the presence of more than 3 polyps and increased risk of EC (Angioni et al., 2008), whereas no trial found any significant correlation with tamoxifen or oestroprogestin intake.

The pathologic characteristics of the non-polypoid endometrium in women with EPs are poorly understood. In the series of Rahimi et al. (2009) including 694 women with typical EPs, the authors found atypical histology in non-polypoid endometrium in the 7.3% of cases (up to 13.2% in postmenopausal patients). In another large trial on 1467 patients, Perri et al. (2010) found EC in the endometrium adjacent to typical EPs in 3.0% of patients. Differently, considerably higher rates of AH and EC were described in the non-polypoid endometrium of women with atypical EP. Mittal and Da Costa (2008) reported a 66% prevalence of AH or EC in the endometrium of patients with atypical EPs undergone hysterectomy. Similarly, Parra et al reported a 66.6% prevalence of EC in the non-polypoid endometrium of a series of women treated for atypical EPs (Parra et al., 2006). These data were confirmed by the two other studies, where the prevalence of EC/AH was estimated in 50% and 88.9%, respectively (Naaman et al., 2015; Elyashiv et al., 2017). Our results agree with the previous findings about the high prevalence of concomitant atypical histology in the non-polypoid endometrium of women with atypical EPs. In this regard, we found concomitantly EC or AH in the 66.6% of patients with atypical EPs undergone hysterectomy. We want to stress that these findings may be of crucial importance for the management of patients with EPs.

Hysteroscopy is an operator dependent technique with high accuracy for the recognition of endometrial lesions in expert hands (Vitagliano et al., 2018). Nevertheless, whilst the majority of atypical lesions (about the 90%) can be identified during hysteroscopy (Garuti et al., 2001; Clark et al., 2002,), a definitive diagnosis can be achieved only by examining EPs and the surrounding non-polypoid endometrium at histopathology. Therefore, for those women with increased risk of atypical EPs, performing multiple biopsies of the non-polypoid endometrium in addition to polypectomy is recommended to exclude the presence of atypical tissue.

## Conclusions

This multicenter trial found a low prevalence of atypical tissue within EPs, especially in premenopausal women. Menopausal status, overweight, age higher than 54 years and EPs size higher that 2 cm were relevant risk factors for atypia.
